# Xmc Mediates Xctr1-Independent Morphogenesis in *Xenopus laevis*†

**DOI:** 10.1002/dvdy.22050

**Published:** 2009-08-03

**Authors:** Tomomi Haremaki, Daniel C Weinstein

**Affiliations:** Biology Department, Queens College of the City University of New YorkFlushing, New York

**Keywords:** Xctr1, Xmc, FGF, Xenopus, morphogenesis

## Abstract

In the frog, *Xenopus laevis*, fibroblast growth factor (FGF) signaling is required for both mesoderm formation and the morphogenetic movements that drive the elongation of the notochord, a dorsal mesodermal derivative; the coordination of these distinct roles is mediated by the *Xenopus* Ctr1 (Xctr1) protein: maternal Xctr1 is required for mesodermal differentiation, while the subsequent loss of Xctr1 promotes morphogenesis. The signaling cascade activated by FGF in the presence of Ctr1 has been well characterized; however, the Xctr1-independent, FGF-responsive network remains poorly defined. We have identified *Xenopus Marginal Coil* (*Xmc*) as a gene whose expression is highly enriched following Xctr1 knockdown. Zygotic initiation of *Xmc* expression in vivo coincides with a decrease in maternal *Xctr1* transcripts; moreover, Xmc loss-of-function inhibits Xctr1 knockdown-mediated elongation of FGF-treated animal cap explants, implicating Xmc as a key effector of Xctr1-independent gastrular morphogenesis. Developmental Dynamics 238:2382–2400, 2009. © 2009 Wiley-Liss, Inc.

## INTRODUCTION

Vertebrate development is driven largely by inductive interactions, during which one group of cells influences the fate of another, often by means of the activity of secreted extracellular ligands. A striking feature of embryonic induction is the prominence of a small set of ligands in these varied interactions, from germ layer formation and early patterning to morphogenesis and organogenesis. This observation raises an important question regarding signal interpretation; that is, how does the embryo ensure that a given signal elicits a response that is specific, and biologically appropriate, to the responding cell? At least two distinct scenarios are plausible. On the one hand, one could envision that a single “core” pathway induced by a given ligand could be modified, at or near the cascade's terminus, by a set of transcriptional effectors specific to one or a few cell types. Alternatively, a single ligand-receptor pair could induce largely distinct signaling cascades, depending upon the cellular context in which the induction takes place.

Members of the fibroblast growth factor (FGF) family play a critical role during innumerable steps of embryogenesis. In the frog, *Xenopus laevis*, FGF is essential for maintaining the differentiation of the mesodermal germ layer, first induced by transforming growth factor-β (TGFβ) signaling (Heasman,^2006^). Shortly after the initiation of gastrulation, FGF receptor activation no longer supports mesodermal differentiation; instead, FGF signaling promotes the dramatic lengthening of both the notochord, a dorsal mesodermal derivative, and the caudal neural tube, by means of the morphogenetic processes of convergence and extension (Nutt et al.,^2001^). FGF mediated-mesoderm induction results from activation of the “canonical” Ras/MAPK cascade (Heasman,^2006^); FGF-mediated morphogenesis, however, results instead from the activation of Ras/MAPK-independent “noncanonical” FGF signaling (Nutt et al.,^2001^). These distinct FGF-dependent pathways are distinguished not only by their activating components, but by their distinct repressors, as well: the *Xenopus* Spred proteins block Ras/MAPK signaling; while the related Sprouty proteins inhibit Ca^2+^ and PKCδ “noncanonical” signaling (Sivak et al.,^2005^). Thus, the shift in the biological consequence of FGF signaling is due to a shift in the signaling cascades stimulated by FGF receptor activation.

A key mechanism by which early embryonic cells “translate” FGF receptor activity into a specific signaling cascade and subsequent biological activity hinges on the presence or absence of the Ctr1 protein within the plasma membrane-associated FGF signaling complex. The maternal *Xenopus* Ctr1 (Xctr1) protein facilitates phosphorylation of the FGF receptor-associated docking protein SNT-1/FRS2α by the FGF receptor and the Laloo nonreceptor tyrosine kinases, as well as the phosphorylation and activation of ERK, the latter event a defining step in “canonical” FGF signaling (Hama et al.,^2001^; Heasman,^2006^; Haremaki et al.,^2007^). Consistent with this biochemical activity, Xctr1 misexpression enhances FGF-mediated mesoderm induction, and inhibits convergent extension, both in explants and in vivo (Haremaki et al.,^2007^). Conversely, loss of Xctr1 inhibits mesoderm differentiation and instead promotes mesodermal and neural morphogenesis (Haremaki et al.,^2007^).

While it is clear that loss of Xctr1 both inhibits differentiation and promotes gastrular morphogenesis, little is yet known of the “noncanonical” signaling cascade that is activated by FGF in the absence of Xctr1. In an attempt to address the mechanisms by which Xctr1 elimination promotes coordinated tissue movements, we performed gene array studies to identify transcripts that were robustly enriched by FGF in the absence of Xctr1. We have identified several genes that are highly expressed following Xctr1 knockdown. One of these, *Xenopus Marginal Coil* (*Xmc*), a gene previously described as an FGF-responsive transcript, was selected for further study (Frazzetto et al.,^2002^). Xmc expression initiates shortly after the initial clearing of maternal *Xctr1* transcripts in the *Xenopus* embryo, and Xmc loss-of-function inhibits Xctr1 knockdown-mediated elongation by FGF, indicating that Xmc is necessary for FGF-stimulated, Xctr1-independent morphogenesis.

## RESULTS

**Fig. 1:**
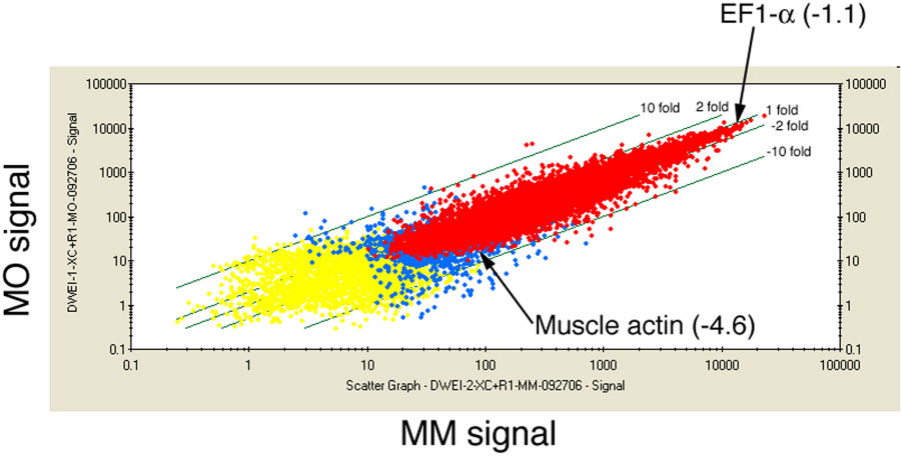
Microarray analysis of Xctr1 knockdown. Data shown are from a representative Affymatrix gene chip hybridization; signal strength was visualized using Affymetrix GeneChip Operating Software 1.4. Transcripts that register hybridization signal with both probes (“Xctr1MO and ”Xctr1MM“) are shown in red. Transcripts that register ”absent“ or ”marginal“ hybridization signal with one probe are shown in blue; transcripts that register ”absent“ or ”marginal“ hybridization signal with both probes are shown in yellow. Examples of unaffected (EF1-α) and down-regulated (Muscle actin) transcripts are highlighted (arrows); number = fold change.

We have previously demonstrated that Xctr1 loss-of-function promotes FGF-mediated elongation of *Xenopus* explant cultures (Haremaki et al.,^2007^). While several signaling components have been implicated in caudal neural and dorsal mesodermal morphogenesis, little is known about the regulation of these events at the level of gene expression. To characterize the transcriptional response to Xctr1 knockdown, we performed microarray analyses to compare the expression profiles of FGF-treated animal caps from embryos injected with either Xctr1-specific (Xctr1MO) or 5-base pair mismatch- (Xctr1MM) morpholinos; Xctr1MM does not inhibit Xctr1 translation, in vitro (Haremaki et al.,^2007^). A representative experiment is shown in Figure 1; the lack of effect on “housekeeping” genes (e.g., *EF1-a*) and the reduction of dorsal mesoderm-specific genes (e.g., *muscle actin*) following Xctr1 knockdown point to the quality of these data sets (Fig. 1; Mohun et al.,^1984^; Krieg et al.,^1989^).

**Fig. 2:**
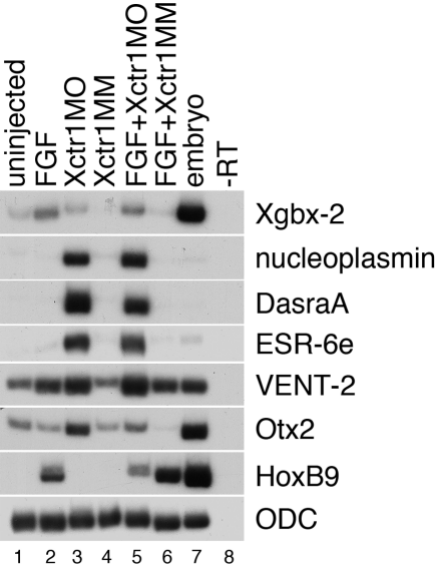
Confirmation of genes with elevated transcription following Xctr1 knockdown. Reverse transcriptase-polymerase chain reaction (RT-PCR) analysis of gene expression in stage 22 animal cap explants. Injection protocol and/or culture conditions are shown at top. A total of 10 ng/ml basic fibroblast growth factor (bFGF) was added, as shown. A total of 250 ng of Xctr1 morpholino (Xctr1MO) and 250 ng of Xctr1 mismatch morpholino (Xctr1MM) were injected, as shown. The induction by HoxB9, a marker of lateral plate mesoderm and spinal cord at this stage, demonstrates that FGF is active in this experiment (Wright et al.,^1990^). The “−RT” lane contains all reagents except reverse transcriptase and was used as a negative control. Ornithine decarboxylase (ODC) is used as a loading control (Bassez et al.,^1990^).

Our aim in these studies was to identify genes whose expression was strongly induced following Xctr1 knockdown. We reasoned that, because both neural and mesodermal differentiation were inhibited by Ctr1 elimination, the genes we identified as “up-regulated” in our analysis were not likely to represent molecular markers of induced cell fates and were, thus, reasonable candidate effectors of Ctr1 knockdown-mediated morphogenesis. We identified 25 annotated genes with greater than threefold elevated expression, following Xctr1 knockdown in FGF-treated neurula stage animal cap explants, in each of two independent microarray experiments (Table 1). Candidates selected for independent verification were confirmed by reverse transcriptase-polymerase chain reaction (RT-PCR; Table 1, bold, Fig. 2, and data not shown). Strikingly, exogenous FGF was not required for enhanced expression of these transcripts; knockdown of Xctr1 was alone sufficient to stimulate this response (Fig. 2).

**Table 1:** Candidate Mediators of Ctrl-Independent Morphogenesis

ID	Symbol	Name	Fold change
**XI.5274.1.S1_at**	DasraA	chromosomal passenger complex protein Dasra A	7.80
**XI.585.1.S1_at**	ESR-6e	enhancer of split related epidermal protein-6	6.74
**XI.1262.1.S1_at**	LOC397921	nucleoplasmin	5.83
**XI.1268.1.S1_at**	Otx2	homeobox protein	4.80
**XI.973.1.S1_at**	LOC397824	homeobox protein Xgbx-2	4.34
**XI.5454.1.S2_at**	Xmc	marginal coil Xmc	4.15
XI.446.1.S1_at	xlZPA	vitelline envelope 69/64 kDa glycoprotein	3.88
XI.5888.2.S1_at	rGAP	rho-GTPase activating protein	3.67
XI.869.1.S2_at	LOC397936	POU-domain protein	3.65
XI.11672.1.A1_at	Otx2	homeobox protein	3.65
XI.798.1.S1_at	B4	histone H1-like maternal protein	3.56
XI.7590.2.A1_at	LOC397951	XDCoH	3.44
**XI.5288.1.S1_at**	LOC398163	cyclin B4	3.42
XI.25540.3.A1_at	LOC398660	KH domain RNA-binding protein Sam68	3.41
**XI.699.1.S1_s_at**	LOC397948	VENT-2 transcription factor	3.41
XI.2582.1.S1_at	rgma	repulsive guidance molecule A protein phosphatase 1D magnesium-dependent, delta	3.31
**XI.8340.2.A1_a_at**	ppm1d	isoform	3.27
XI.15540.2.A1_at	atf5	activating transcription factor 5	3.23
XI.8719.1.S1_at	dynII2	dynein light chain 2	3.20
XI.17481.1.A1_at	mthfd2	methylene tetrahydrofolate dehydrogenase (NAD+dependent), methenyltetrahydrofolate cyclohydrolase	3.13
XI.15540.1.A1_at	atf5	activating transcription factor 5	3.13
**XI.1592.1.A1_at**	LOC397824	homeobox protein Xgbx-2	3.12
XI.8701.1.S1_a_at	aldoc	aldolase C, fructose-bisphosphate	3.11
XI.7590.2.A1_x_at	LOC397951	XDCoH	3.07
XI.23306.1.S1_at	cldn6	claudin 6	3.05

^a^List of *Xenopus laevis* transcripts with expression up-regulated at least threefold in fibroblast growth factor (FGF)-treated animal cap explants from embryos injected with a Ctrl-specific morpholino (XctrlMO). Only annotated genes are listed.

**Fig. 3:**
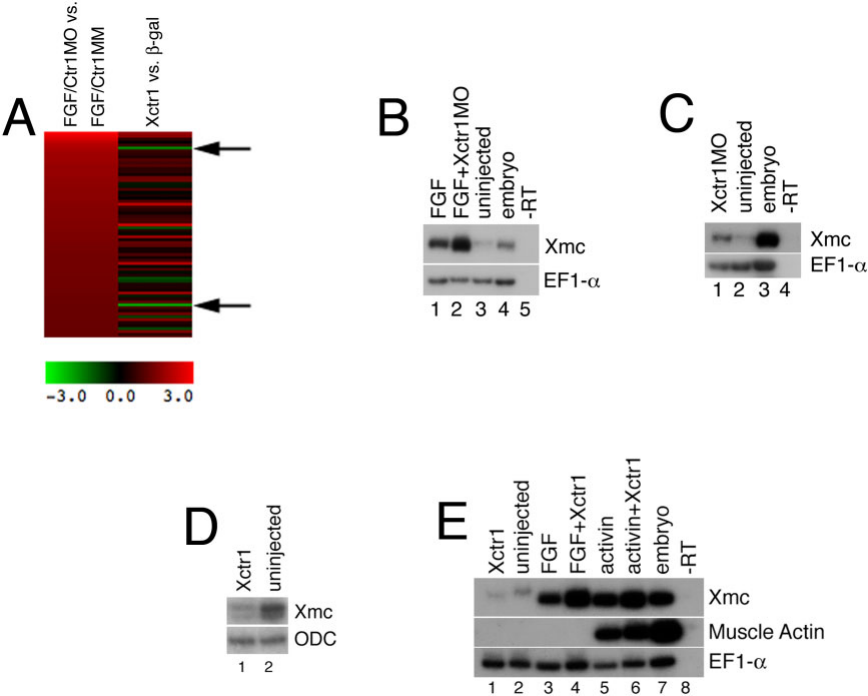
Regulation of Xmc by Xctr1. **A**: *Xmc* expression is suppressed by Xctr1. Microarray analysis of gene expression in embryos injected with RNA encoding either *Xctr1* or *β-galactosidase*. Left column: genes that are expressed more highly in fibroblast growth factor (FGF) treated caps taken from Xctr1MO injected embryos than in FGF treated caps taken from Xctr1MM injected embryos. Right column: Relative expression of genes from left column in animal caps from embryos injected with either *Xctr1* or *β-gal* RNA. Up-regulated genes are shown in red; down-regulated genes are shown in green. Arrows point to two transcripts that are strongly down-regulated by Xctr1 (top: 38%, bottom: 28% of signal in β-galactosidase-injected control samples); both transcripts encode Xmc. B–E: Reverse transcriptase-polymerase chain reaction (RT-PCR) confirmation of microarray data presented in A. **B**: *Xmc* expression is elevated following Xctr1 knockdown in FGF-treated animal caps. **C**: *Xmc* expression is elevated following Xctr1 knockdown in animal caps cultured in saline. **D**: *Xmc* expression is suppressed following Xctr1 misexpression in animal caps. **E**: Xctr1 does not inhibit *Xmc* induction by either FGF or Activin protein. Ectopic Xctr1 inhibited Activin-mediated cap elongation in these assays (data not shown). A total of 10 ng/ml bFGF, 0.5 ng/ml Activin was added, as shown. A total of 250 ng of Xctr1 morpholino (Xctr1MO) and 250 ng of Xctr1 mismatch morpholino (Xctr1MM) were injected, as shown. Also, 1 ng of *Xctr1* and 1 ng of *β-galactosidase* RNA was injected, as shown.

To narrow the pool of candidate effectors, we reasoned that mediators of Xctr1-independent morphogenesis might also show a reduction in transcript abundance following Xctr1 misexpression, as Xctr1 inhibits elongation of both dorsal mesoderm and caudal neurectoderm (Haremaki et al.,^2007^). We therefore performed an additional microarray study to select for transcripts down-regulated by Xctr1 in animal cap explants. Figure 3A shows the effects of Xctr1 misexpression on transcripts shown to be up-regulated in the Xctr1 knockdown microarray studies. Of the transcripts enriched following injection of Xctr1 morpholino oligos (Fig. 3A, left panel, red), only two were significantly down-regulated following misexpression of Xctr1 RNA (Fig. 3A, right panel, arrows pointing to bright green bands). Both of these bands correspond to *Xenopus marginal coil* (*Xmc*). The FGF-inducible *Xmc* gene encodes a coiled-coil protein previously implicated in the regulation of gastrulation movements (Frazzetto et al.,^2002^); it was thus chosen for further analysis. RT-PCR was used to confirm that Xctr1 knockdown enhances Xmc expression in the presence of FGF (Fig. 3B). *Xmc* transcripts are also enriched following Xctr1 knockdown in the absence of FGF (Fig. 3C). We next confirmed, using RT-PCR, that Xctr1 misexpression down-regulates *Xmc* in animal caps (Fig. 3D,E, lanes 1 and 2). FGF protein induces robust expression of Xmc in animal cap explants, presumably reflecting mesodermal Xmc expression; Activin protein induces levels similar to those seen with FGF (Fig. 3E, compare lanes 2, 3, and 5; Frazzetto et al.,^2002^). Ectopic Xctr1 does not, however, inhibit *Xmc* expression in either FGF- or Activin-treated caps (Fig. 3E). These data demonstrate that Xctr1 loss-of-function enhances *Xmc* expression in both ectoderm and mesoderm, and that Xctr1 gain-of-function suppresses Xmc expression in ectoderm, but not mesoderm.

**Fig. 4:**
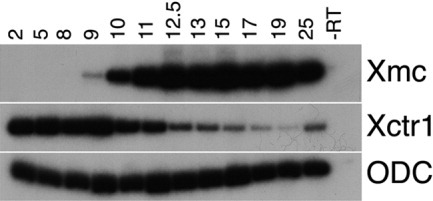
Reverse transcriptase-polymerase chain reaction (RT-PCR) analysis of *Xmc* expression during early *Xenopus* development. Xmc expression is zygotic, and coincides with diminishment of maternal Xctr1 transcripts.

Our studies suggest that Xctr1 knockdown may promote morphogenesis by means of induction of Xmc. Consistently, we find that initiation of Xmc expression coincides with the decline in maternal *Xctr1* expression (Fig. 4; Frazzetto et al.,^2002^; Haremaki et al.,^2007^). Coupled with the gain- and loss-of-function analyses described above, these correlative data provide further evidence for regulation of Xmc by Xctr1, in vivo.

**Fig. 5:**
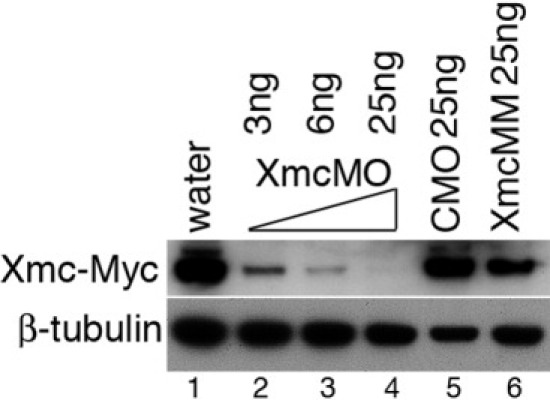
XmcMO inhibits translation of 3′ Myc-tagged *Xmc* RNA (Xmc-Myc) containing the XmcMO binding site. Xmc-Myc translation is not inhibited by co-injection of a five base pair mismatch Xmc morpholino (XmcMM), or a control scrambled morpholino (CMO). Western blot of protein lysates from stage 10 *Xenopus* embryos, injected as listed, using anti-Myc and anti-β-tubulin antibodies, as shown (Sigma).

We observed no effects on cell fate determination or morphogenesis from Xmc misexpression in ectodermal or mesodermal explants, including animal caps treated with FGF and/or Activin, or in intact *Xenopus* embryos, consistent with earlier published studies (data not shown; Frazzetto et al.,^2002^), suggesting that Xmc gain-of-function is not sufficient to alter morphogenesis. We next sought to address the potential requirement for Xmc in mediating Xctr1-independent morphogenesis. Knockdown of Xmc function by means of Xmc-specific antisense morpholino oligonucleotides has been shown to affect axial elongation in *Xenopus* (Frazzetto et al.,^2002^); morpholino studies were thus used to further address the relationship between Xmc and Xctr1 during morphogenesis. Injection of an Xmc-specific morpholino (XmcMO) results in a dose-dependent translational block of a co-expressed Myc epitope-tagged Xmc construct (Xmc-Myc) in gastrula stage embryos (Fig. 5; Frazzetto et al.,^2002^). This effect cannot be mimicked with either a control, “scrambled” morpholino (CMO), or with an Xmc-specific morpholino containing 5 base pair mismatches (XmcMM; Fig. 5). Injection of XmcMO has been previously shown to generate three phenotypic classes by the tadpole stage: embryos with a kinked axis, embryos with a short axis, and embryos with an open blastopore (Class I, II, and III, after Frazzetto et al.,^2002^). In our hands, dorsal marginal injection of 25 ng of XmcMO at the four-cell stage results in 87% of embryos with Class II defects, with the remainder exhibiting Class III defects (N = 15). This dose of XmcMO was used for subsequent Xmc loss-of-function studies.

**Fig. 6:**
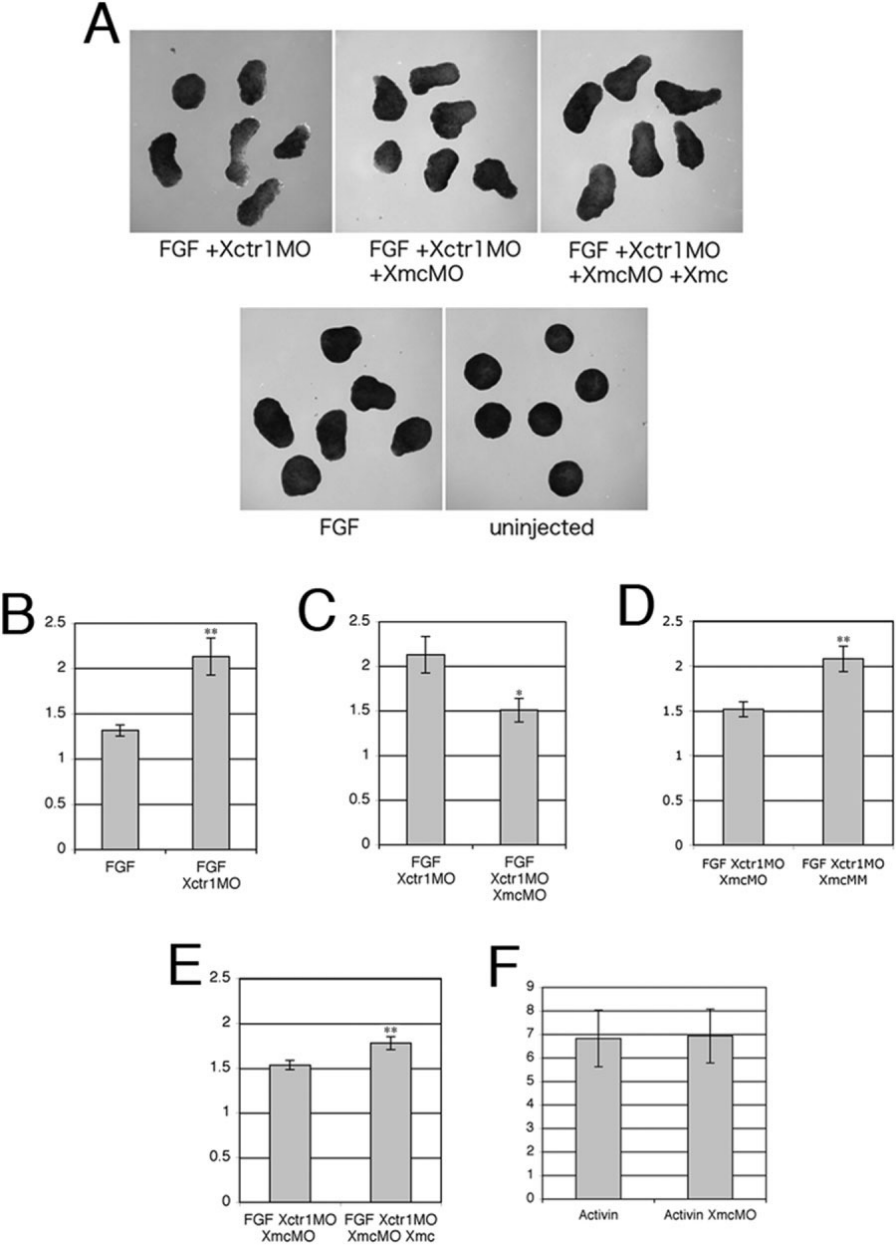
Effects of Xmc loss-of-function on explant morphogenesis. **A**: Injection of morpholinos against Xmc (XmcMO) inhibits Xctr1 knockdown-mediated morphogenesis in fibroblast growth factor (FGF) -treated animal cap explants at stage 22; this effect is rescued by co-expression of *Xmc* RNA (Xmc) that lacks the XmcMO binding site. B–E: Quantification of the requirement for Xmc on explant morphogenesis. Error bars represent the standard error of the mean. y-axis = ratio of cap length:cap width. **B**: Xctr1 knockdown increases elongation of FGF-treated animal caps. For FGF, N = 17; for FGF/Xctr1MO, N = 19, with N = number of caps assayed. **C**: Xmc knockdown inhibits Xctr1 knockdown-mediated elongation. For FGF/Xctr1MO, N = 19; for FGF/Xctr1MO/XmcMO, N = 18. **D**: XmcMM does not inhibit Xctr1 knockdown-mediated elongation. For FGF/Xctr1MO/XmcMO, N = 25; for FGF/Xctr1MO/XmcMM, N = 26. **E**: Suppression of Xctr1 knockdown-mediated elongation by XmcMO is rescued by co-expression of *Xmc* RNA lacking the XmcMO binding site (Xmc). For FGF/Xctr1MO/XmcMO, N = 58; for FGF/Xctr1MO/XmcMO/Xmc, N = 57. **F**: Xmc knockdown does not affect Activin-mediated elongation. For Activin, N = 8; for Activin/XmcMO, N = 9. *P < 0.005; **P < 0.01. A total of 10 ng/ml bFGF, and 0.5 ng/ml Activin was added, as shown. Also, 2 ng of *Xmc* RNA, 250 ng of Xctr1MO, 25 ng of XmcMO, and 25 ng of XmcMM were injected, as shown.

Explant assays were used to test for the potential requirement for Xmc in Xctr1 knockdown-mediated morphogenesis. Elongation of FGF-treated animal cap explants is enhanced following Xctr1 knockdown (Fig. 6A,B) (Haremaki et al.,^2007^); this elongation is significantly inhibited by co-injection of the Xmc-specific morpholino XmcMO (Fig. 6A,C). Inhibition of Xctr1 knockdown-mediated elongation is not seen after injection of a 5 base pair mismatch Xmc morpholino (XmcMM; Fig. 6D); moreover, the block to inhibition by XmcMO can be partially rescued by co-injection of *Xmc* RNA lacking the XmcMO binding site (Xmc), pointing to the specificity of XmcMO for the *Xmc* transcript in these assays (Fig. 6A,E). Xmc knockdown does not, however, inhibit elongation of animal caps by Activin (Fig. 6F); thus, Xmc is required for FGF-mediated, Xctr1-independent morphogenesis.

## DISCUSSION

The presence or absence of the transmembrane protein Ctr1 dictates the interpretation of FGF receptor activation as, respectively, either a pro-differentiation or a pro-morphogenesis cue. Here, we demonstrate that Xmc is a critical component of the signaling cascade activated by FGF in the absence of Xctr1, as Xmc loss-of-function inhibits Xctr1 knockdown-mediated morphogenesis. This functional link is buttressed by the temporal patterns of expression of the two genes: the initiation of *Xmc* expression at gastrula stages correlates with the initial decline in maternal *Xctr1* expression levels, and also with the initiation of FGF-mediated morphogenesis.

Xmc represents one of more than two dozen genes whose expression is induced following Xctr1 knockdown; it was selected for further analysis because it was the only transcript that was also suppressed following Xctr1 misexpression in ectodermal explants. We have found, however, that Xctr1 misexpression, despite inhibiting explant and embryo elongation, does not block Xmc expression in the presence of FGF or Activin (this study, and Haremaki et al.,^2007^). This suggests that, although Xmc is required for Xctr1-independent, FGF-mediated elongation, Xmc suppression does not underlie Xctr1-dependent inhibition of mesodermal elongation; early suppression of Xmc may, however, play a role in the inhibition of neural morphogenesis by Xctr1. Other candidates from our screens, perhaps including factors that are not required for mediating Xctr1-independent morphogenesis, may be involved in the suppression of premature mesodermal morphogenesis downstream of Xctr1 (Haremaki et al.,^2007^); we are beginning to test, in gain- and loss-of-function assays, microarray targets whose expression is altered by *Xctr1* RNA, but not Xctr1 morpholino, injection.

## EXPERIMENTAL PROCEDURES

### Gene Chip Analysis

RNA from 80 animal cap explants, cultured to stage 20, were used to generate hybridization probes for use on Affymetrix GeneChip *Xenopus laevis* Genome Arrays; hybridization was performed with the help of the Mount Sinai Microarray Shared Research Facility (http://www.mssm.edu/research/resources/microarray/). One nanogram of *Xctr1* or *β-galactosidase* RNA, 250 ng of Xctr1MO or 250 ng of Xctr1MM morpholinos were injected, as described. Microarray data were normalized by RMA (Irizarry et al.,^2003^) and analyzed using the affylmGUI Bioconductor package (Wettenhall et al.,^2006^). Normalized data were visualized by MeV4.0 (Saeed et al.,^2003^).

### Xmc Cloning and Mutant Construction

An Image clone containing the *Xenopus marginal coil* (*Xmc*) gene was obtained from Open Biosystems (IMAGE ID: 6634521; accession no. BC072122). The full coding sequence of Xmc was amplified by PCR and cloned into the *Eco*RI and *Xho*I sites of pCS2++. For Xmc-Myc, 16 base pairs of 5′-untranslated region (UTR) and the coding sequence of Xmc was subcloned upstream of six Myc (EQKLISEEDLNEM) epitopes in the CS2+MT vector; this construct contains the complete XmcMO binding site. The Xmc construct used for morpholino rescue experiments lacks all 5′-UTR and thus much of the XmcMO binding site.

### RNA Preparation, Explant Dissection, and Cell Culture

RNA was synthesized in vitro in the presence of cap analog using the mMessage mMachine kit (Ambion). Microinjection, explant dissection, and cell culture were performed as described (Hemmati-Brivanlou and Melton,^1994^; Wilson and Hemmati-Brivanlou,^1995^).

### RT-PCR

RT-PCR was performed as described (Wilson and Hemmati-Brivanlou,^1995^). Primers used in this study are as follows: Xgbx2-U: 5′-CCCTTGACAGATTAGATCGG; Xgbx2-D: 5′-CTCGCACTATACTTCTGTCC; Nucleoplasmin-U: 5′-AGCCTAAGCGAGTTGCTTTG; Nucleoplasmin-D: 5′-TAGACTGGCAATCGGAACTG; DasraA-U: 5′-ACTACTGCTCTAACCAGGTC; DasraA-D: 5′-CTGTATCTCCTCTCAGTAGC; ESR-6e-U: 5′- GGCACAGGGCAATACTGGT; ESR-6e-D: 5′-CCCCACTTGGCATTATGTTC; VENT-2-U: 5′-ACCCACTAATGGAAACCCTG; VENT-2-D: 5′-CACATGGCCCAATATTAGCC; Otx2-U: 5′-CGGGATGGATTTGTTGCA; Otx2-D: 5′- TTGAACCAGACCTGGACT; Xmc-U: 5′-CTGGTGTTACAGACCAAGGGG; Xmc-D: 5′-ACCTGTGCTTTTGCCACTC; Xctr1-U: 5′-GTGTGTCTGCTGGAAACTAAG; Xctr1-D: 5′-TTCCCGCGAAATCTTCAG; ODC-U: 5′-AATGGATTTCAGAGACCA; ODC-D: 5′-CCAAGGCTAAAGTTGCAG.

### Morpholinos

Morpholino oligos (Gene Tools) were heated for 5 min at 65°C, then quenched on ice, before injection at the two- or four-cell stage. XmcMO: TGCAGC(CAT)TATGTATATGTAAAAA; Xmc MM: TGgAGC(gAT)TATcTAaATcTAAAAA. Additional morpholino sequences are as described (Frazzetto et al.,^2002^; Haremaki et al.,^2007^).
